# Toxicities of CAR-T, Bispecific Antibodies, and Antibody–Drug Conjugates in Multiple Myeloma: A Practical Approach to Risk Mitigation and Management

**DOI:** 10.3390/cancers18132083

**Published:** 2026-06-26

**Authors:** Sereen Hej-Ali, Kyle Banwell, Halima Mohamed, Andrea Cervi, Adina Dass, Rasna Gupta, Caroline Hamm, Sindu Kanjeekal, Ian Strange Seguel, Morgan Szalay, Sahar Khan

**Affiliations:** 1 Department of Biomedical Science, Faculty of Science, University of Windsor, Windsor, ON N9B 3P4, Canada; 2 Windsor Regional Hospital, Windsor, ON N8W 1L9, Canada; 3 Department of Oncology, Schulich School of Medicine and Dentistry, Western University, London, ON N6A 5W9, Canada

**Keywords:** multiple myeloma, CAR-T cell therapy, bispecific antibodies, antibody–drug conjugate, BCMA, GPRC5D, cytokine release syndrome, ICANS, toxicity management, risk stratification

## Abstract

B-cell maturation antigen (BCMA), G protein-coupled receptor class C group 5 member D (GPRC5D)-directed immunotherapies, chimeric antigen receptor T-cell (CAR-T) products, bispecific T-cell engagers (BsAbs), and antibody–drug conjugates (ADCs), have transformed the management of MM. Their adoption is now extending beyond tertiary centers following FDA modifications for CAR-T safety and the rapid uptake of off-the-shelf bispecifics suitable for community delivery. Clinicians outside specialist hubs must therefore be conversant with the full toxicity spectrum, including rare but high-consequence events, both for informed consent and for the work-up of post-therapy complications. In this narrative review, we report on the published literature around toxicities of approved and investigational BCMA- and GPRC5D-directed therapies, drawing on pivotal trial data, real-world cohorts, pharmacovigilance studies, and consensus management recommendations, with emphasis on practical recognition and risk mitigation. This review presents toxicities by a temporal pattern including acute (CRS, ICANS, infection, ocular, mucocutaneous), subacute (cranial nerve palsies, parkinsonism, myelitis, peripheral neuropathies IEC-associated enterocolitis and cardiovascular events), and long-term (prolonged cytopenias, second primary malignancies). We discuss validated risk stratification tools, such as the CAR-HEMATOTOX score, EASIX index, and multidisciplinary geriatric assessment, which predicts severe ICANS, infection, and resource utilization, supporting individualized pre-treatment planning. Safe delivery of immune therapies in community settings requires infrastructure for acute critical care, neurology, ophthalmology, infectious disease and long-term surveillance, but is achievable when paired with validated risk stratification and clear referral pathways.

## Simple Summary

Multiple myeloma (MM) is a cancer of plasma cells that is currently being treated with powerful new immune therapies, including CAR-T cell products, bispecific antibodies, and antibody–drug conjugates. These therapies have demonstrated responses and efficacy in relapsed disease; however, they cause a wide range of side effects. Some of these appear immediately; including fevers, low blood pressure, and confusion which are driven by therapeutic overactivation of the immune system. Others develop over weeks or months, including serious infections, movement disorders, gut inflammation, prolonged drops in blood counts, and rare second cancers. Until recently, these treatments were largely confined to specialist hospitals. However, new regulatory changes across North America and the rapid uptake of bispecific antibodies have expanded their delivery into community settings. This review summarizes the toxicities clinicians should anticipate, offers a practical framework for managing them, and outlines the infrastructure smaller centers need.

## 1. Introduction

Multiple myeloma (MM) is often recognized as the second most common hematologic malignancy globally, yet despite therapeutic progress with proteasome inhibitors, immunomodulatory drugs, and anti-CD38 monoclonal antibodies, most patients eventually relapse. The arrival of B-cell maturation antigen (BCMA)-directed immune therapies has reshaped the relapsed/refractory landscape, achieving overall response rates of 60–95% in heavily pre-treated populations [1]. BCMA is a plasma cell-restricted surface protein overexpressed on malignant cells, making it an ideal therapeutic target [2,3,4]. Dominant modalities include chimeric antigen receptor T-cell (CAR-T) therapy, in which a patient’s own T-cells are genetically engineered to express a synthetic anti-BCMA receptor, and bispecific antibodies/T-cell engagers (BsAbs/BiTEs), which are off-the-shelf antibodies that bridge CD3 on T-cells to BCMA on plasma cells [5,6,7].

FDA-approved products of these therapies feature two CAR-T agents (idecabtagene vicleucel and ciltacabtagene autoleucel) and three approved BsAbs (teclistamab, elranatamab, linvoseltamab). Belantamab mafodotin, an antibody–drug conjugate (ADC), provides an additional BCMA-focused option with a distinctive toxicity profile [8]. Meanwhile, GPRC5D-directed therapies, such as talquetamab, were approved in 2023, and other constructs (CAR-T, BsAbs, trispecific antibodies) continue to be in clinical development [9,10,11,12,13]. Pivotal efficacy data for these agents are summarized in Table 1 (approved therapies) and Table S1 (in-development).

Recent regulatory developments have amplified the clinical relevance of toxicity management, as FDA modifications to the requirements of the Risk Evaluation and Mitigation Strategy (REMS) for several CAR-T products now permit its administration outside designated centers. Furthermore, the approval and increasing real-world uptake of bispecific antibodies, amenable to outpatient delivery once initial step-up dosing is complete, is rapidly expanding the population of oncologists who will encounter these agents and their toxicities [14]. This narrative review was developed to provide a practical, clinically oriented overview of the recognized toxicities associated with BCMA- and GPRC5D-directed therapies in multiple myeloma, including chimeric antigen receptor T-cell (CAR-T) therapies, bispecific antibodies, and antibody–drug conjugates. The review focuses on the recognition, work-up, risk stratification, prevention, and management of treatment-related adverse events relevant to informed consent discussions and post-therapy care. We also cover distinct, less familiar, sometimes delayed complications that smaller centers must be equipped to recognize. Relevant publications were identified through targeted searches on the PubMed database and review of key references, encompassing foundational and contemporary studies published between 2004 and 2026. We conclude with a framework for infrastructure development and multidisciplinary support that are required to deliver these therapies safely.

**Table 1:** Clinical efficacy data for FDA-approved BCMA- and GPRC5D-targeted multiple myeloma therapies.

Class	Agent	Trial	Phase	N	Efficacy
BCMA CAR-T	Ide-cel	KarMMa, NCT03361748	2	100	ORR: 72%, ≥VGPR: 53% [15]
	Cilta-cel	CARTITUDE-1, NCT03548207	1/2	97	ORR: 97.9%, ≥VGPR: 94.9% [16]
BCMA × CD3 BsAb	Tec	MajesTEC-1, NCT03145181	1/2	110	ORR: 61.8%, ≥VGPR: 57.3% [17]
	Elranatamab	MagnetisMM-3, NCT04649359	2	123	ORR: 61%, ≥VGPR: 56.1% [18]
	Linvoseltamab	LINKER-MM1, NCT03761108	1/2	221	ORR: 60.2%, ≥VGPR: 52.0% [19]
GPRC5D × CD3 BsAb	Tal	MonumenTAL-1, NCT03399799/ NCT04634552	1/2	375	ORR: 70.7%, ≥VGPR: 58.2% [9]
BCMA ADC	Belamaf	DREAMM-2, NCT03525678	2	221	ORR: 35.7%, ≥VGPR: 21.7% [20]

Abbreviations: BCMA, B-cell maturation antigen; CAR-T, chimeric antigen receptor T-cell therapy; GPRC5D, G protein-coupled receptor class C group 5 member D; BsAb, bispecific antibody; ADC, antibody–drug conjugate; ORR, overall response rate; VGPR, very good partial response; Ide-cel, idecabtagene vicleucel; Cilta-cel, ciltacabtagene autoleucel; Tal, talquetamab; Tec, teclistamab.

## 2. Acute Toxicities (Days–Weeks)

### 2.1. CRS and ICANS

The pathophysiology of cytokine release syndrome (CRS) and immune effector cell-associated neurotoxicity syndrome (ICANS) is defined by a hyperinflammatory cascade initiated when T-cell redirection therapies (listed in Table 1) activate effector cells, triggering the release of primary cytokines such as IFN-γ and TNF-α [21,22,23]. CRS and ICANS rates, biomarkers, and risk features across currently available T-cell redirection therapies are summarized in Table 2. The primary cytokines stimulate bystander cells, such as macrophages and monocytes, to secrete a cascade of secondary mediators, including IL-6, IL-1, IL-8, and IL-10 [21,22,23,24]. IL-6 is the central driver of systemic CRS symptoms, promoting vascular leakage, hypotension, and fever by activating endothelial cells [21,23,24]. ICANS occurs when systemic inflammation disrupts the blood–brain barrier (BBB), allowing cytokines and leukocytes to infiltrate the central nervous system and activate resident microglia [21,23,24,25,26]. In the acute setting, corticosteroids remain the first-line intervention; while tocilizumab is the cornerstone of CRS management, its use in ICANS is reserved for grade 2+ cases concurrent with CRS, as IL-6 receptor blockade may paradoxically worsen neurotoxicity by elevating systemic IL-6 levels [27]. For steroid-refractory ICANS, the IL-1 receptor antagonist anakinra represents a viable second-line option.

### 2.2. Infections

Although the benefits of treatment with BsAbs are significant, patients undergoing these treatments face an increased risk of adverse events (AEs), including infections [14]. In a 165-patient, phase I/II teclistamab study (MajesTEC-1), 76.4% of patients experienced infection AEs of any grade [31]. In another phase II study, including 123 patients on elranatamab, the MagnetisMM-3 study, 61.8% reported infections of any grade, and 31.7% experienced high-grade infections [42]. In the MonumenTAL-1 study with talquetamab, only 57% of patients experienced any grade infections, and only 16.8% experienced a grade 3/4 toxicity, in those receiving the lower dose [43,44]. A systematic review and meta-analysis on infections following bispecific antibodies in myeloma found that the overall risk of infections with BsAb therapy is estimated to be approximately 56% for all grades, with grade 3 or 4 infections occurring in 24% of cases [34,45]. Those patients treated with BsAbs in combination with other therapies, 71%, were also observed to have significantly higher rates of all-grade infection than those receiving bispecific monotherapies, 52%.

The high infection risk of these treatments is often even higher in patients who meet the criteria for neutropenia or hypogammaglobulinemia (HGG) [45]. For those with a 20% higher risk of developing febrile neutropenia, the use of colony-stimulating factors (CSFs) is suggested during the first treatment cycle [45]. If neutropenia is chronic or prolonged despite G-CSF treatment attempts, antibacterial or anti-fungal prophylaxis should be considered [14]. In HGG, replacement therapy with intravenous immunoglobulins is suggested if patients’ IgG levels fall below 400 mg/dL [34,45], particularly in patients with history of infection. There were very high rates of both neutropenia and hypogammaglobulinemia in the previously mentioned teclistamab study with all grade rates being 71% and 75%, respectively. The rates of high-grade neutropenia were particularly high with 64% of patients experiencing grade 3 or higher [46]. HGG and neutropenia are recognized risk factors for infections. Neutrophils are a key part of the body’s defense system against infections, which is why infection risk is associated with the severity and duration of neutropenia [45]. A large-scale cohort study of patients treated with CD20xCD3 BsAbs identified neutropenia and hypogammaglobulinemia as significant independent risk factors for infections complications. Analysis revealed that neutropenia increased the hazard for all-grade infections by over six-fold, while hypogammaglobulinemia was a potent predictor for both all-grade and severe grade 3–5 infectious episodes. These findings highlight the importance of regular monitoring and management of these immune conditions to mitigate the high cumulative incidence of infections seen in patients undergoing BsAb therapy [35].

There are also many prophylactic vaccination recommendations for patients undergoing BsAbs treatment. It is recommended that patients follow the general guidelines on the use of live attenuated vaccines, though it should be considered that live vaccines are not suggested in MM patients [14]. It is also suggested that post-stem cell transplant vaccinations be carried out before starting BsAb treatment, where feasible. Given the increased risk of infection among patients with MM treated with BsAbs, a range of prophylactic strategies have been suggested across the literature. These recommendations are summarized in Supplementary Table S2.

Regarding CAR T therapies, the U.S. prescribing information (USPI) for ide-cel includes warnings of prolonged cytopenia, hypogammaglobulinemia, and serious infections. In the CART ide-cel study mentioned previously, of the 222 recipients, 39% experienced prolonged neutropenia and 37% experienced prolonged thrombocytopenia. Infections rates were also very high in those receiving ide-cel, with 56% percent experiencing any grade of infection and 20% experiencing grade 3 or higher infection. Both of these were lower than the rates reported for the standard of care treatment for RRMM [39]. Cilta-cel, another CAR T therapy, appears to have higher rates of infection when compared with ide-cel in heavily pre-treated patients [40].

### 2.3. Ocular Toxicities

Ocular toxicities maintain a heavier visibility in the anti-BCMA ADCs literature, with studies describing adverse events that are early-onset, typically emerging within days to weeks of initial infusion [47,48]. In a real-world safety and efficacy study of the toxicity profile of Belantamab Mafodotin, ophthalmic adverse events (grade ≤ 2) were the most common toxicity reported at 48% [49]. Although the exact mechanisms underlying ocular toxicities remain unknown, one proposed explanation for ADCs is an off-target uptake of monomethyl auristatin F (MMAF) payload by corneal epithelial cells via macropinocytosis, leading to intracellular toxicity [48,50,51,52]. Recent pharmacovigilance studies indicate the relevance of these toxicities in other BCMA modalities, as exemplified by a systematic review of the FDA Adverse Event Reporting System (FAERS) conducted by Frey et al. (2024), which identified ~53 ocular adverse events across CAR-T therapies [53]. In CAR-T, the mechanism behind ocular toxicity development is thought to be indirect and immune-mediated, driven by intense cytokine release and inflammation, which can affect ocular and blood barriers [47]. This may help explain the generally lower rates of ocular adverse events (~2%) reported in CAR-T studies [47]. Management of this toxicity was shown in the literature to be most effective through dose modification, including delaying, reducing, or extending the dosing interval [54]. In the literature by expert panels across North Africa, Latin America, and the Middle East, practical recommendations for management include screening with an ophthalmologist before treatment to determine risk, ongoing reassessment once treatment is started, and cross-disciplinary collaborations between the hematologist and ophthalmologist to determine changes to dosing level/interval depending on severity, with interruption recommended for more serious developments such as keratitis [55,56]. The articles highlight that, as seen in the DREAM 7 and 8 clinical trials, changes in therapy administration with belantamab mafodotin did not significantly impact efficacy [55,57]. Although mitigative measures exist, such as prophylactic steroid eye drops, studies such as DREAMM-2, a clinical trial detailed in Table 1, have shown that these measures are ineffective [51,58].

### 2.4. Skin and Nail Toxicity

Skin and nail toxicities are among the most characteristic adverse events associated with GPRC5D-targeted therapies; skin toxicities reported in approximately 60–80% and nail-related toxicities in up to around 50–55% of patients treated with agents such as talquetamab across clinical studies (including MonumenTAL-1) [59,60,61]. These toxicities typically emerge early in the treatment course, often within the first few weeks or cycles, and manifest as rash, pruritus, dry or exfoliative skin, palmar-plantar erythrodysesthesia-like changes, and a spectrum of nail abnormalities including discoloration, ridging, onycholysis, and nail fragility [59,60,61]. Mechanistically, the leading hypothesis is an on-target, off-tumor effect driven by GPRC5D expression in normal tissues, particularly within hair follicles, eccrine glands, and the keratogenous zone of the nail, resulting in immune-mediated damage to these structures following T-cell engagement [12,43,59,60,62,63,64,65,66]. Management is largely supportive and symptom-directed, with emollients and topical corticosteroids forming the mainstay for skin toxicities, and escalation to systemic corticosteroids reserved for persistent or higher-grade cases; nail toxicities are typically managed with moisturizers, topical steroids, protective measures (e.g., gloves, nail care), and avoidance of trauma, with adjuncts such as vitamin E oil or biotin used in practice [43,44,59,60,61,62,67,68]. Importantly, while these toxicities are generally low grade and rarely lead to discontinuation, dose modifications (delays, reductions, or interruptions) have been effective in improving symptoms in more bothersome cases [43,59,60].

## 3. Subacute and Delayed Toxicities (Weeks–Months)

### 3.1. Neurologic Toxicities Beyond ICANS

#### 3.1.1. Cranial Nerve Palsies

One of the most common non-ICANS neurologic toxicities associated with BCMA-directed CAR T-cell therapy, with an incidence of approximately 6–9% reported across clinical trials of ciltacabtagene autoleucel (cilta-cel) and supported by multiple cohort analyses, is cranial nerve palsies [69,70,71,72]. These events typically occur in the early to subacute period, around 22 days post-infusion, and most commonly involve isolated cranial nerve deficits, particularly facial nerve (seventh cranial nerve) palsy [70,72,73]. Management is generally effective and follows approaches similar to idiopathic facial nerve palsy, with short courses of corticosteroids (e.g., prednisolone) representing the mainstay of treatment [70,74]. In clinical studies, corticosteroid use resulted in resolution in approximately 90% of cases, with a median recovery time of 66 days [70,72]. Supportive care and evaluation of alternative etiologies, including infection or leptomeningeal disease via MRI and cerebrospinal fluid analysis, are essential, and overall prognosis is favorable, with most cases demonstrating full reversibility [70,75].

#### 3.1.2. Parkinsonism

Among BCMA-directed CAR T-cell therapies for MM, delayed movement and neurocognitive toxicities (MNTs), particularly parkinsonism, represent a distinct subset of non-ICANS neurologic toxicities [73,76]. These events occur in approximately 1–5% of patients treated with ciltacabtagene autoleucel (cilta-cel) [69,77,78]. These toxicities typically emerge weeks to months after infusion, presenting with bradykinesia, rigidity, bradyphrenia, flat affect, personality changes, apathy, gait disturbance, and cognitive impairment [76,78]. Mechanistically, the leading hypothesis involves off-tumor/on-target effects, whereby CAR T cells traffic to BCMA-expressing regions of the central nervous system, particularly dopaminergic neurons in the basal ganglia, resulting in neuroinflammation and neuronal dysfunction [75,76,78]. Management of delayed Parkinsonism following BCMA-directed CAR T-cell therapy remains challenging and poorly standardized, with limited responsiveness to conventional immunosuppressive approaches [70,75,79]. Pharmacokinetic monitoring may aid early detection, as absolute lymphocyte counts within 10–14 days post-infusion can reflect CAR T-cell expansion and help identify patients at increased risk for delayed toxicities [75,80,81]. Standard Parkinson’s disease treatments such as levodopa are generally ineffective, supporting a distinct immune-mediated pathophysiology [76]. Emerging evidence suggests targeted immunomodulation, including JAK inhibitor ruxolitinib, may improve immunotherapy-induced parkinsonism [82]. Despite these approaches, interruption or discontinuation of immunotherapy remains an option, if feasible, to potentially regain stabilization or improvement, although neurologic recovery is often incomplete and prolonged [75].

#### 3.1.3. Myelitis

Within BCMA-directed CAR T-cell therapy for MM, myelitis represents an exceedingly rare manifestation of non-ICANS neurologic toxicities, which overall occur in approximately 0.8% of patients [70,73,75,78,83]. When it occurs, myelitis typically presents days to weeks after infusion and manifests as acute or subacute spinal cord dysfunction, including weakness, sensory deficits, paraplegia, and autonomic dysfunction such as urinary retention, reflecting inflammatory injury to the spinal cord [78]. In some cases, infectious triggers such as HHV-6 reactivation may further contribute to spinal cord inflammation [75,83,84]. Management remains largely extrapolated from autoimmune and inflammatory myelitis, with high-dose intravenous corticosteroids as first-line therapy, often leading to partial neurologic and radiographic improvement [70,78,83,84]. In refractory or severe cases, additional immunomodulatory strategies including IVIG, plasmapheresis, anakinra, or anti–IL-6 therapy (e.g., siltuximab) have been used with variable success, although evidence is limited to case reports and no standardized treatment approach has been established [70,78,83].

#### 3.1.4. Peripheral Neuropathies

Among non-ICANS neurologic toxicities following BCMA-directed CAR T-cell therapy, peripheral neuropathies constitute an uncommon but clinically important manifestation, with a pooled incidence of approximately 0.8% and peripheral neuropathy accounting for roughly 7.5% of reported NINT events in a large meta-analysis of 4630 patients [73]. Guillain–Barré syndrome (GBS) and related polyneuropathies typically present in a delayed fashion, occurring days to weeks after CAR T infusion, often after resolution of CRS and ICANS, and manifest as ascending weakness, areflexia, and varying degrees of sensory and autonomic dysfunction consistent with acute immune-mediated peripheral nerve injury [70,73,85,86]. Management is largely extrapolated from standard treatment of immune-mediated neuropathies and other CAR T-related neurotoxicities, with high-dose corticosteroids commonly used as first-line therapy and representing the most consistently reported intervention [73,78,87]. In more severe or refractory cases, IVIG and plasmapheresis have been used based on case reports and small series, particularly in GBS-like presentations, although responses are variable and evidence remains limited [87].

### 3.2. Immune Effector Cell-Associated Enterocolitis

The recent literature has reported immune effector cell-associated enterocolitis (IEC-EC), a rare, delayed toxicity following CAR-T therapy characterized by non-bloody diarrhea, abdominal pain, and inflammatory patterns resembling those seen in graft-versus-host disease (GVHD), beginning a median of 92.5 days after therapy initiation [88]. A retrospective multicenter analysis across 11 centers reported an overall incidence of 1.2%, with agent-specific rates of 0.2% for idecabtagene vicleucel and 2.2% for ciltacabtagene autoleucel [88]. Somay et al. (2025) further suggest that IEC-EC may involve pre-existing intestinal autoimmune T-cell clones that expand after CAR T therapy, cross-reactivity of CAR T cells with intestinal epithelial antigens, or secondary enterocyte damage from nearby activated T-cells as potential mechanisms [89]. In discussing the management of IEC-EC, the literature stresses the importance of ruling out infectious and neoplastic causes of diarrhea before making a diagnosis of IEC-EC [88,90]. Generally, in early stages, corticosteroids can be used, although the literature is variable on their effectiveness. If symptoms do not see resolution shortly, it is recommended to minimize steroid use and assign biologic agents such as infliximab or vedolizumab. Should clinical improvement continue to be scarce, it could be evidence of a T-cell lymphoproliferative process, and the diagnosis must be revisited with consideration of more effective drugs such as cyclosporine [88,90].

### 3.3. Cardiovascular Toxicity

A pharmacovigilance study by Zhang et al. (2024) conducted a disproportionality analysis of the FAERS database to understand the emergence of cardiovascular toxicities in MM patients undergoing therapy [91]. The study identified 3,228 cases of adverse events with 95.3% of patients experiencing serious consequences that included hospitalization and death. The study further identified an agent-dependent component to cardiovascular AEs, with insignificant safety signals observed for belantamab (IC025/ROR025 = −1.23/0.44) and teclistamab (IC025/ROR025 = −0.82/0.59), whereas significant disproportionality signals were observed for daratumumab (IC025/ROR025 = 0.26/1.20) and elotuzumab (IC025/ROR025 = 0.34/1.28) [91]. Manifestation of this toxicity in the literature includes cardiomyopathy, tachycardia, arrhythmias, cardiac failure, hypotension, ischemic heart disease, and thrombosis, among others [91,92,93]. For anti-BCMA CAR T-cell therapy, the literature on cardiovascular adverse events indicates a strong association with CRS. KarMMa, a phase 2 clinical trial, reported hypotension in 16% of 128 patients who received ide-cel [94]. Meanwhile, in a retrospective cohort study of 137 CAR-T-receiving patients, 17 cardiovascular events (i.e., arrhythmias, decompensated heart failure, death) were reported, of which 18% occurred in the 11 MM patients [95]. CRS levels were 84% and 59%, respectively, with the latter study testing the association between adverse events and CRS, finding that grade ≥ 3 CRS occurred in 50% of patients (*n* = 6) compared with 11% with mild or no CRS (*n* = 131) [94,95]. Further, the adverse event itself matters, as in a retrospective cohort study of 211 CAR T-treated patients with MM or lymphoma, cardiovascular adverse events were most frequently observed within the first month following treatment, yet a subset of 15.2% of events were observed to have actually developed ≥12 months [96]. Standardized protocols are limited; therefore, management of cardiovascular toxicities typically includes pretreatment risk assessment, mitigation of CRS through IL-6 antagonists (e.g., Tocilizumab) or corticosteroids, and close cardiac monitoring [97,98].

## 4. Long-Term Toxicities (Months–Years)

### 4.1. Prolonged Cytopenia

Cytopenias are arguably among one of the more common toxicities of CAR-T therapy, with a variability of incidence in the literature. In a retrospective study and experimental analysis of 48 patients with RRMM, an overall incidence of 95.7% across grades of cytopenia was reported in CART patients, with a further breakdown of anemia (97.9%), neutropenia (97.9%), and thrombocytopenia (77%) [99]. Conversely, a clinical trial evaluating prolonged toxicities of CAR-T therapy in 54 RRMM patients found rates of severe cytopenia that included neutropenia (52%), severe anemia (28%), and thrombocytopenia (33%) [100]. The mechanism leading to prolonged cytopenias in CAR-T is not fully understood; however, it is considered to be multifactorial, with proposed causes such as prior/baseline impact on blood count due to MM disease or previous treatment, lymphodepletion, persistent immune activation following CAR-T heightening cytokine expression, and disruption of bone marrow microenvironment and hematopoietic differentiation due to inflammatory cytokines promoting apoptosis [99,101,102]. Management of this toxicity often involves G-CSF with the expectation of gradual improvement over weeks, with recommendations of a bone marrow biopsy or aspirate if progress is elusive [103,104]. This is particular to the case of prolonged cytopenia, as there may be an underlying diagnosis of immune effector cell-associated hemophagocytic lymphohistiocytosis-like syndrome (IEC-HS), where the use of steroids or anakinra should be considered [104]. For specific cytopenias (e.g., thrombocytopenia), a thrombopoietin mimetic drug may be initiated, such as eltrombopag [103,104].

### 4.2. Second Primary Malignancies

A recent systematic review by Agha et al. (2026) that included 17 studies around MM with a total of 2154 patients found that SPMs occurred in a median of 5.9% of patients treated with CAR-T [105]. Another systematic review and meta-analysis of 25 studies, including 5517 patients, reported a pooled estimate of SPMs at 6.0% among the subset of MM patients (n = 1362) following CAR-T treatment [106]. In November 2023, the FDA issued a communication identifying T-cell lymphomas as a concerning complication of CAR-T [107,108]. A FAERS pharmacovigilance study by Di Napoli et al. (2024) reported 17 cases of developed T-cell malignancies, of which 70.6% were T-cell lymphomas and 41.2% were fatal, with disproportionate reporting signals observed for axicabtagene ciloleucel and tisagenlecleucel [109]. Studies specific to MM describe lymphocytosis and erythematous facial lesions, with a case report by Braun et al. (2025) reporting a symptomatic peripheral T-cell lymphoma 9 months post-ciltacabtagene autoleucel in a 63-year-old patient [107,110,111]. Multiomic profiling demonstrated hyperexpanded CAR-positive T-cell clones with TET2-mutated clonal evolution [112]. Although specific guidelines for managing SPMs remain limited, the available literature generally emphasizes the importance of long-term monitoring [113,114].

## 5. Patient Selection and Risk Stratification

### 5.1. Patient Selection for Intensive Therapies

Despite high clinical efficacy of intensive therapies (i.e., BCMA-directed CAR T-cell therapy and bispecific antibodies (BsAbs)), these treatments are associated with significant toxicities, including CRS, ICANS, and prolonged hematotoxicity. As such, patient selection must look beyond disease status to include factors such as disease tempo, functional reserve, organ function, and marrow reserve [115].

### 5.2. Role of Frailty Assessments and Risk-Stratification Assessments

Frailty, a multifactorial state encompassing physical function, cognition, comorbidities, polypharmacy, and social support, is prevalent among RRMM patients, with up to 61% identified as frail at the time of CAR-T infusion [116]. Although frailty has been associated with inferior PFS and OS relative to non-frail counterparts [116,117,118], baseline frailty measures do not consistently predict high-grade CRS or ICANS [116,119], indicating that chronological age and conventional vulnerability markers should not serve as automatic exclusion criteria for intensive therapy [120,121]. Accordingly, frailty assessments are best utilized for individualized risk stratification and treatment planning, as multidisciplinary geriatric evaluations can identify modifiable risk factors, such as polypharmacy, which has been independently associated with ICANS development rather than as a basis for rigid therapeutic exclusion [119,122].

The practical utility of frailty assessments is further enhanced when integrated with quantitative risk stratification tools, namely the Endothelial Activation and Stress (EASIX) index (creatinine × LDH/platelets) and the CAR-HEMATOTOX (HT) score [27,123,124], which are summarized in Table 3. In RRMM patients, a high HT score (≥2) is a potent predictor of severe ICANS (16% vs. 0% in HT-low patients, *p* < 0.001), an independent predictor of severe infections (aOR 4.9, *p* = 0.03), and is associated with prolonged hospitalization (13 vs. 8 days) [27,124]. Notably, both tools demonstrate high negative predictive value (NPV), rendering them particularly effective for ruling out risk of early, severe complications [124].

Risk stratification using these scores enables a proactive approach to ICANS management, in which high-risk patients may benefit from low-dose prophylactic corticosteroids (e.g., dexamethasone), which have been shown to reduce severe neurologic toxicity without detrimental effects on response rates or CAR T-cell expansion.

## 6. Implications for Smaller Centers

Recent alterations to the regulatory landscape of immunotherapies have increased the likelihood of community oncologists encountering rarer toxicities associated with MM treatments. Substantial infrastructure is required to deliver these therapies safely, which is achievable when paired with validated risk stratification and clear referral pathways.

Acute care infrastructure must support 24 h monitoring during the high-risk CRS/ICANS window. At minimum, this requires inpatient beds with continuous vital sign monitoring, immediate availability of tocilizumab and dexamethasone (with anakinra accessible within hours for steroid-refractory ICANS), rapid laboratory turnaround for ferritin, CRP, fibrinogen, and triglycerides, and a clearly defined escalation pathway to intensive care for vasopressor-dependent hypotension or grade 3+ ICANS. Centers without on-site ICU coverage should establish formal transfer agreements with a tertiary partner before initiating therapy.

Multidisciplinary support must be available for prompt work-up and management of potential adverse effects. Neurology should be available for the work-up of delayed cranial nerve palsies, parkinsonism, myelitis, and peripheral neuropathies, including same-week access to MRI and lumbar puncture. Ophthalmology partnership is essential for centers delivering belantamab mafodotin, with pre-treatment screening, ongoing slit-lamp surveillance, and capacity for dose-modification decisions. Infectious diseases input is needed for the management of atypical opportunistic infections, particularly in patients receiving sequential T-cell-redirecting therapies.

Risk-stratified patient pathways operationalize the literature reviewed above. The CAR-HEMATOTOX score and EASIX index should be calculated pre-treatment for all CAR-T candidates, with high-risk patients (HT score ≥ 2 or EASIX > 2.15) flagged for prophylactic dexamethasone consideration and prolonged monitoring. Multidisciplinary geriatric assessment should be standard for patients aged ≥70 or with frailty markers, recognizing that chronological age alone is not an exclusion criterion, and that geriatric impairments frequently improve in responders. Centers uncertain about specific candidates should retain a low threshold for tertiary consultation.

Patients require structured long-term follow-up with particular attention to cytopenias persistent beyond three months, second primary malignancy surveillance (e.g., rare but reportable risk of T-cell lymphoma) and delayed cardiovascular events. Consideration should also be given to rarer complications such as IEC-EC, which can occur months after therapy and may present first to primary care or other specialists unfamiliar with these complications. Establishing a designated post-CAR-T or post-bispecific clinic, even if low-volume, may help provide a structured framework for surveillance.

## 7. Limitations

Several considerations should be taken into account when interpreting the findings presented in this review. As a narrative review, it does not employ a formal systematic review methodology and may therefore be subject to selection bias. In addition, many uncommon toxicities, particularly delayed neurologic, gastrointestinal, and target-specific adverse events, are supported primarily by retrospective studies, pharmacovigilance reports, small case series, and individual case reports. The review focuses primarily on currently approved BCMA- and GPRC5D-directed therapies; with the aim of providing a practical framework for recognizing and managing toxicities associated with agents currently available in clinical practice. However, the treatment landscape is rapidly evolving toward dual-targeting CAR-T constructs, trispecific antibodies, combination bispecific approaches, sequential immune redirection strategies and alternative cellular platforms such as CAR-NKT therapies. As clinical experience with these emerging therapies remains limited, their toxicity profiles, including the potential for cumulative toxicities following sequential CAR-T and bispecific antibody exposure, are not yet fully characterized and warrant further investigation. Consequently, some recommendations presented herein are based on the best available evidence and expert consensus rather than prospective comparative data.

## 8. Conclusions

BCMA- and GPRC5D-directed immune therapies have produced remarkable response rates in relapsed/refractory multiple myeloma, but their toxicity spectrum extends far beyond cytokine release syndrome and immune effector cell-associated neurotoxicity. Clinicians must remain vigilant for delayed neurologic syndromes including cranial nerve palsies, parkinsonism, myelitis and peripheral neuropathies; for IEC-associated enterocolitis and cardiovascular events occurring weeks to months after therapy; and for long-term complications such as prolonged cytopenias and second primary malignancies, including the rare but reportable risk of CAR-positive T-cell lymphoma. Validated risk stratification tools, the CAR-HEMATOTOX score, EASIX index, and multidisciplinary geriatric assessment, should inform individualized pre-treatment planning rather than serve as rigid exclusion criteria, and CHIP screening shows promise as a future biomarker. With FDA REMS modifications and the expansion of bispecific antibody use into outpatient settings, the safe delivery of these therapies in community centers is achievable when paired with the multidisciplinary infrastructure, risk-stratified pathways, and long-term surveillance frameworks outlined here.

## Figures and Tables

**Table 2:** CRS and ICANS rates by agent.

Therapy Target Protein	Infection Rate All Grades Grade ≥ 3	Cytopenias Rate All Grades Grade ≥ 3	CRS Rate All Grades Grade ≥ 3	ICANS Rate All Grades Grade ≥ 3	Pathophysiology & Risk Insights
Talquetamab (GPRC5D) [9,28,29,30]	26–76% (All) 17–62% (≥G3)	2–69% (All) 3–47% (≥G3)	49–80% (All) 0.7–2.1% (≥G3)	4–11% (All) 0.7–4.1% (≥G3)	B-cell sparing mechanism that leads to lower rates of high-grade infection. Can cause “on-target off-tumor” toxicities (dysgeusia, skin rashes, nail dystrophy).
Teclistamab (BCMA) [31,32]	76.4–78% (All) 44.8–62%	40–72% (All) 21.2–65% (≥G3)	72% (All) 0.6% (≥G3)	3–15% (All) 0–2.4% (≥G3)	Binds CD3 to promote malignant cell destruction. Prior exposure to T-cell redirection therapy reduces CRS.
Elranatamab (BCMA) [18,33,34,35]	56–69.9% (All) 35–44% (≥G3)	48.8–78% (All) 22–67% (≥G3)	56–57.7% (All) 0% (≥G3)	3.4–11% (All) 0% (≥G3)	Biphasic pattern. Clinical response is uniquely correlated with the expansion rate of CD8+ T-cells post-infusion rather than baseline counts.
Cilta-cel (BCMA) [36,37]	58% (All) 20% (≥G3)	79–96% (All) 60–95% (≥G3)	95% (All) 4% (≥G3)	17% (All) 2–9% (≥G3)	High incidence of CRS, events are mainly low-grade.
Ide-cel (BCMA) [35,36,38,39,40]	69% (All) 22% (≥G3)	16–91% (All) 16–99% (≥G3)	84% (All) 5% (≥G3)	18% (All) 3% (≥G3)	Use of the 4-1BB costimulatory domain generally results in slower expansion and lower CRS severity.
Linvoseltamab (BCMA) [19,41]	71–75% (All) 28.9–48% (≥G3)	8.6–44% (All) 31.5–72.7% (≥G3)	45.3–46% (All) 0.9–1% (≥G3)	7.3–7.7% (All) 0.49–2.6% (≥G3)	Facilitates the cytolytic killing of MM cells by redirecting CD3+ effector T-cells to target BCMA-expressing plasma cells.

Abbreviations: CRS, cytokine release syndrome; ICANS, immune effector cell-associated neurotoxicity syndrome. Ranges reflect cross-study variability.

**Table 3:** Risk stratification models. Summarizes validated tools for predicting toxicity and survival in myeloma patients. EASIX is preferred for its simplicity and use of widely available markers to mirror disease burden and endothelial stress. CAR-HT has a very high NPV, making it exceptionally effective for “ruling out” patients who are at low risk for early, severe complications. GA-MDC is the most effective at identifying patients who will require prolonged hospitalization (31 vs. 17 days) or ICU care.

Model/Study	Population	Scores and Measures	Predictivity and Statistical Data
CAR-HEMATOTOX (HT) [27,124]	CAR-T ((113) RRMM patients)	ANC, Hemoglobin, Platelets, CRP, Ferritin.	AUC of 0.82 for severe neutropenia; high NPV (rule out risk).
		Risk of prolonged neutropenia (≥14 days), severe ICANS, and OS.	HT^high^ patients had 16% severe ICANS vs. 0% in HT^low^ (*p* < 0.001). OS was significantly shorter (median 10.5 mo vs. NR, *p* < 0.001).
GA-MDC Recommendation [125]	CAR-T (61 evaluated; 53 treated, 11 BCMA myeloma)	Multidisciplinary geriatric assessment (functional, cognitive, nutritional, etc.).	The consensus recommendation was a strong independent predictor of OS and resource use.
		Suitability for CAR-T (“Proceed” vs. “Decline”).	BCMA “Proceed” patients had superior OS (16.4 vs. 4.2 mo, *p* = 0.03). “Decline” patients had ICU rates of 50% vs. 6% in the “Proceed” group (*p* = 0.01).
Simplified Frailty Index (SFI) [116,120]	CAR-T: 821 RRMM in Akhtar; 136 in Davis.	Age, ECOG performance status, HCT-CI.	Significant predictor of any grade ICANS and OS.
		ICANS, infection, and survival (PFS/OS).	Frail patients had higher rates of ICANS (37% vs. 21.5%, *p* < 0.01) and inferior PFS (6.9 vs. 11.1 mo, *p* = 0.028).
INPI/GPS [118,119,125]	CAR-T (139 RRMM patients)	Primarily, CRP and Albumin.	“Highly predictive of survival” even when adjusting for high-risk disease status.
		Impact of immuno- nutritional status on survival in RRMM.	Inflammatory markers (i.e., CRP > 3) are independently associated with significantly shorter median OS (8.6 vs. 17.3 mo, *p* = 0.03).
EASIX/ Modified EASIX [123]	CAR-T (across multiple disease cohorts, including RRMM)	(Creatinine × LDH)/Platelets; Modified adds CRP and Ferritin.	High EASIX (>2.15) predicts a five-fold higher risk of severe ICANS (19% vs. 4%) and near-identical increase in overall mortality hazard (HR 4.85).
		Endothelial activation and risk of severe CRS/ICANS.	

Abbreviations: ANC = absolute neutrophil count; AUC = area under the curve; INPI = Inflammatory Prognostic Index; GPS = Glasgow Prognostic Score; NPV = negative predictive value; OS = overall survival; PFS = progression-free survival; NR = not reached; HCT-CI = Hematopoietic Cell Transplantation Comorbidity Index; ECOG = Eastern Cooperative Oncology Group.

## Data Availability

No new data was generated for this review. All analyzed data was cited within the article.

## Supplementary Materials

The following supporting information can be downloaded at https://www.mdpi.com/article/10.3390/cancers18132083/s1. Supplemental Table S1. Clinical efficiency data for in-development BCMA- and GPRC5D-targeted multiple myeloma therapies. Supplemental Table S2. Summary of prophylactic recommendations for patients with MM receiving bispecific antibodies. References [126,127,128,129,130,131,132,133,134,135,136,137,138,139,140,141] are cited in the Supplementary Materials.

## Abbreviations

ADC, antibody–drug conjugate; AE, adverse event; ANC, absolute neutrophil count; AUC, area under the curve; BBB, blood–brain barrier; BCMA, B-cell maturation antigen; BsAb, bispecific antibody; CAR-T, chimeric antigen receptor T-cell; CHIP, clonal hematopoiesis of indeterminate potential; cilta-cel, ciltacabtagene autoleucel; CRS, cytokine release syndrome; ECOG, Eastern Cooperative Oncology Group; EASIX, Endothelial Activation and Stress Index; FAERS, FDA Adverse Event Reporting System; G-CSF, granulocyte colony-stimulating factor; GBS, Guillain–Barré syndrome; GPRC5D, G protein-coupled receptor class C group 5 member D; HCT-CI, Hematopoietic Cell Transplantation Comorbidity Index; HGG, hypogammaglobulinaemia; HT, CAR-HEMATOTOX; ICANS, immune effector cell-associated neurotoxicity syndrome; ide-cel, idecabtagene vicleucel; IEC-EC, immune effector cell-associated enterocolitis; IEC-HS, immune effector cell-associated hemophagocytic lymphohistiocytosis-like syndrome; IVIG, intravenous immunoglobulin; MM, multiple myeloma; MMAF, monomethyl auristatin F; NPV, negative predictive value; ORR, overall response rate; OS, overall survival; PFS, progression-free survival; REMS, Risk Evaluation and Mitigation Strategy; RRMM, relapsed/refractory multiple myeloma; SIR, standardized incidence ratio; SPM, second primary malignancy; VGPR, very good partial response.
