# Supplementary material for: Toxicities of CAR-T, Bispecific Antibodies, and Antibody–Drug Conjugates in Multiple Myeloma: A Practical Approach to Risk Mitigation and Management
Source: Cancers (Basel). 2026 Jun 26;18(13):2083. doi: 10.3390/cancers18132083 (PMC13359683; doi:10.3390/cancers18132083)
Supplement: Supplementary file 1 [file cancers-18-02083-s001.zip › cancers-4360556-supplementary.pdf]

Supplementary Material

Toxicities of CAR-T, Bispecific Antibodies, and Anti-Body Drug Conjugates in Multiple Myeloma: A Practical Approach to Risk Mitigation and Management

Sereen Hej-Ali [1,†*], Kyle Banwell [1†], Halima Mohamed [1], Andrea Cervi[2,3], Adina Dass [1], Rasna Gupta [2,3], Caroline Hamm[2,3], Sindu Kanjeekal [2,3], Ian Seguel Strange [1], Morgan Szalay [1], Sahar Khan [2,3]

Supplemental Table S1. Clinical efficacy data for in-development BCMA & GPRC5D-targeted multiple myeloma therapies.

Class	Target	Agent	Trial	Phase	N	Efficacy	Infections	Toxicities
BsAb	GPRC5D x CD3	Forimtamig [62,126]	BP42233, NCT04557150	1	120	ORR: 66.7%, ≥VGPR: 54.2% [126]	46.7% (All), 21.9% (≥ G3)	Cytop: NR (All), Anemia: 9.3%, TCP: 16.2%, NP: 14.3% (≥ G3); CRS: 80.0% (All), 1.9% (≥ G3); ICANS: 8.6% (All), 1.9% (≥ G3)
	GPRC5D x CD3	LBL-034 [127]	NCT06049290	1/2	55	ORR: 70.9%, ≥VGPR: 56.4%	NR	Cytop: NR (All), LYMP: 51.8%, NP: 28.6%, LEUK: 25.0%, TCP: 17.9%, Anemia: 16.1% (≥ G3); CRS: 73.2% (All), 1.8% (≥ G3); ICANS: NR (All), NR (≥ G3)
	BCMA x CD3	Etentamig [128]	NCT03933735; NCT05650632	1/1b	146	ORR: 66%, ≥VGPR: 54%	NR (All), 22% (≥ G3)	Cytop: NR (All), NP: 38%, Anemia: 23%, LYMP: 25%, TCP: 16% (≥ G3); CRS: 30% (All), 0% (≥ G3); ICANS: NR (All), NR (≥ G3)
	FcRH5 x CD3	Cevostamab [129]	GO39775; NCT03275103	1/2	167	ORR: 43.1%, ≥ VGPR: 25.7%	53.9% (All), 19.2% (≥ G3)	Cytop: NR (All), NP: 31.1% (All), 28.2% (≥ G3), Anemia: 23.4% (All), 18.0% (≥ G3); CRS: 74.3% (All), 1.8% (≥ G3); ICANS: 13.2% (All), 1.8% (≥ G3)
	BCMA x CD3	REGN5459 [130]	NCT04083534	1/2	43	ORR: 67%, ≥ VGPR: 58%	61% (All), 37% (≥ G3)	Cytop: NR (All), NP: 37% (All), 37% (≥ G3), Anemia: 35% (All), 26% (≥ G3), LYMP: 23%, TCP: 19% (≥ G3); CRS: 54% (All), 5% (≥ G3); ICANS: 2% (All), 0% (≥ G3)
	BCMA x CD3	EMB-06 [131]	NCT04735575	1/2	40	ORR: 92%, ≥ VGPR: 75	30% (All), NR (≥ G3)	Cytop: NR (All), NP: 30% (All), NR (≥ G3), LEUK: 25% (All), NR (≥ G3), Anemia: 25% (All), NR (≥ G3), LYMP: 25% (All), NR (≥ G3), TCP: 17.5% (All), NR (≥ G3); CRS: 25% (All), 0% (≥ G3); ICANS: 0% (All), 0% (≥ G3)

BsAb + Combination	GPRC5D × CD3 + CRBN/IMiD	Tal + Pom [132]	MonumenTAL-2, NCT05050097	1	35	ORR: 84.8%, ≥VGPR: 71.8%	71.4% (All), 22.9% (≥ G3)	Cytop: NR (All); NP: 60.0% (All), 48.6% (≥ G3); Anemia: NR (All), 25.7% (≥ G3); TCP: NR (All), 20.0% (≥ G3); CRS: 74.3% (All), 2.9% (≥ G3); ICANS: 5.7% (All), 0% (≥ G3)
	GPRC5D × CD3 + CD38	Tal + Dar [133]	TRIMM-2, NCT04108195	1	65	ORR: 80.0%, ≥VGPR: 69.2%	71% (All), 29% (≥ G3)	Cytop: 75% (All), 62% (≥ G3/4), Anemia: 26%, NP: 29%, TCP: NR (≥ G3); CRS: 78% (All), 0% (≥ G3); ICANS: 5% (All), 0% (≥ G3)
TsAb	GPRC5D × CD3 + BCMA × CD3	Tal + Tec [134]	RedirecTT-1, NCT04586426	1/2	90	ORR: 79.0%, ≥VGPR: NR	NR (All), 31% (≥ G3)	Cytop: NR (All), NR (≥ G3); CRS: 78% (All), NR (≥ G3); ICANS: NR (All), NR (≥ G3)
	BCMA × GPRC5D × CD3	Ramantamig [13]	NCT05652335	1	124	ORR: 73.0%, ≥VGPR: 66.0%	75% (All), 28% (≥ G3)	Cytop: NR (All), NP: 48% (All), 41% (≥ G3); CRS: 59% (All), 0% (≥ G3); ICANS: 2% (All), 0% (≥ G3)
	BCMA x CD38 x CD3	ISB 2001 [135]	TRIgnite-1 / NCT05862012	1	14	ORR: 75%, ≥ VGPR: 25.0%	14.3% (All), 7.1% (≥ G3)	Cytop: NR (All), NR (≥ G3); CRS: 71.4% (All), 0% (≥ G3); ICANS: 0% (All), 0% (≥ G3)
CAR T	GPRC5D	CT071 [136]	NCT05838131	1	20	ORR: 100%, ≥VGPR: 70%	NR (All), 15% (≥ G3)	Cytop: NR (All), TCP: 10% (≥ G3); CRS: 60% (All), 0% (≥ G3); ICANS: 5% (All), 5% (≥ G3)
	GPRC5D	MCARH109 [67,80]	NCT04555551	1	17	ORR: 71%, ≥VGPR: 59%	18% (All), 12% (≥ G3)	Cytop: NR (All), NP: 94%, TCP: 65%, Anemia: 35% (≥ G3); CRS: 88% (All), 6% (≥ G3); ICANS: 6% (All), 6% (≥ G3)
	GPRC5D	Arlo-cel [137]	QUINTESSENTIAL, NCT06297226	2	79	ORR: 87%, ≥VGPR: NR	50% (All), 17% (≥ G3)	Cytop: NR (All), NR (≥ G3); CRS: 82% (All), NR (≥ G3); ICANS: 10% (All), 2% (≥ G3)
	GPRC5D	RD118 [138]	NCT05219721/ NCT05759793	1	18	ORR: 94.4%, ≥VGPR: 88.9%	94.4% (All), 55.6% (≥ G3)	Cytop: NR (All), LEUK: 100% (All), 94.4% (≥ G3), LYMP: 100% (All), 100% (≥ G3), Anemia: 83.3% (All), 50.0% (≥ G3), TCP: 94.4% (All), 77.8% (≥ G3), NP: 100% (All), 100% (≥ G3); CRS: 88.9% (All), 5.6% (≥ G3); ICANS: 5.6% (All), 5.6% (≥ G3)
	BCMA x CD19	AZD0120 [139]	DURGA-1 / NCT05850234	1/2	25	ORR: 100%, ≥ VGPR: 80%	NR	Cytop: NR (All), NP: 56% (All), 52% (≥ G3), Anemia: 32% (All), NR (≥ G3), LYMP: NR (All), 32% (≥ G3), LEUK: NR (All), 32% (≥ G3); CRS: 64% (All), 0% (≥ G3); ICANS: 0% (All), 0% (≥ G3)
	BCMA	Anito-cel [140]	iMMagine-1 / NCT05396885	2	117	ORR: 97%, ≥ VGPR: NR	NR (All), 9% (≥ G3)	Cytop: NR (All), NP: 66%, Anemia: 24%, TCP: 24% (≥ G3); CRS: 85% (All), 1% (≥ G3); ICANS: 8% (All), 1% (≥ G3)

Abbreviations: BCMA, B-cell maturation antigen; CAR-T, chimeric antigen receptor T-cell therapy; GPRC5D, G protein-coupled receptor class C group 5 member D; BsAb, bispecific antibody; Ide-cel, idecabtagene vicleucel; Cilta-cel, ciltacabtagene autoleucel; Tal, talquetamab; Tec, teclistamab. ORR, overall response rate; VGPR, very good partial response; Cytop, cytopenias; TCP, thrombocytopenia; NP, neutropenia; CRS, cytokine release syndrome; LYMP, lymphopenia; LEUK = leukopenia; ICANS, immune effector cell-associated neurotoxicity syndrome; NR, not reported

Supplemental Table S2. Summary of prophylactic recommendations for patients with MM receiving bispecific antibodies

Infections	Prophylaxis Recommendations
Viral Infections	
General	• Before treatment, patients should be screened for HBV, HCV, HIV, and COVID-19 as well as resolving any active or uncontrolled infections [141].<br>• Acyclovir or valacyclovir should be used as protection against HSV and VZV reactivation in all RRMM patients [14,142].<br>• This prophylactic treatment should be administered indefinitely regardless of a patient's vaccination status, and the MM treatment should be maintained during its administration [45].<br>• Monitoring is not recommended while using the prophylactic treatments above [14].
VZV	• Acyclovir or valacyclovir [14,141].<br>• Vaccination against VZV is recommended in RRMM patients
HBV	• If a patient is core antibody positive, prophylaxis should be administered, or they should be monitored for HBV DNA copies.<br>• For those with positive DNA tests, antiviral treatment is suggested [14].<br>• If a patient is surface antigen positive, administer antiviral prophylaxis according to a specialist's advice and standard treatment guidelines, including entecavir, tenofovir, and lamivudine [14,141].
Influenza	• It is recommended that patients receiving BsAbs and their close contacts receive the influenza vaccination [14].<br>• The vaccination should include a two-dose series of high-dose influenza vaccine at least one month apart.

SARS-COV-2	• Vaccination recommendations from the CDC or the local health authority should be followed<br>• If effective prophylactic antibodies are available for the prevalent variant, treatment with these mAbs is recommended [14].
Bacterial Infection	• Prophylaxis recommendations in patients with: [14]<br>o Prolonged neutropenia<br>o High risk of infections<br>o History of recurrent bacterial infections<br>• Based on NCCN guidelines, treatment with levofloxacin should start with BsAb treatment, continue through the first cycle, and go on until the patient no longer has neutropenia [14,45].<br>• Risk of developing resistant pathogens should be considered with the use of anti-bacterial prophylaxis, as the use of antibacterial prophylaxis in the absence of neutropenia is a point of controversy [45].<br>• Combining anti-bacterial prophylactic treatments is not recommended [14].
Fungal Infections	
General	• Only recommended if the patient has: [14,141]<br>o Previous history of fungal infections<br>o Prolonged neutropenia<br>o History of prolonged high-dose corticosteroid use (<2 weeks)<br>• Administer prophylaxis via the direction of an infectious disease specialist if available: [14]<br>o Fluconazole is recommended<br>o Itraconazole and voriconazole can be considered<br>o Monitoring during anti-fungal prophylaxis is not recommended except in special cases
P. jirovecii pneumonia (PJP)	• Recommended prophylaxis for all patients due to its high mortality of 30-60% [45,141].<br>• Trimethoprim-sulfamethoxazole is suggested<br>• If a patient is allergic to sulfonamide, alternatives are dapsone or atovaquone<br>• Patients with neutropenia should consider inhaled or intravenous pentamidine

Abbreviations*: HBV = Hepatitis B Virus; HCV = Hepatitis C Virus; HIV = Human Immunodeficiency Virus; HSV = Herpes Simplex Virus; VZV = Varicella Zoster Virus; RRMM*

= Relapsed and Refractory Multiple Myeloma; BsAbs = Bispecific antibodies; CDC = Centers for Disease Control; mAbs = Monoclonal Antibodies, NCCN = National Comprehensive Cancer Network
